# Supplementary material for: Nuciferine Inhibits Oral Squamous Cell Carcinoma Partially through Suppressing the STAT3 Signaling Pathway
Source: Int J Mol Sci. 2023 Sep 26;24(19):14532. doi: 10.3390/ijms241914532 (PMC10572883; doi:10.3390/ijms241914532)
Supplement: Supplementary file 1 [file ijms-24-14532-s001.zip › ijms-2614838-supplementary.pdf]

Supplementary Material

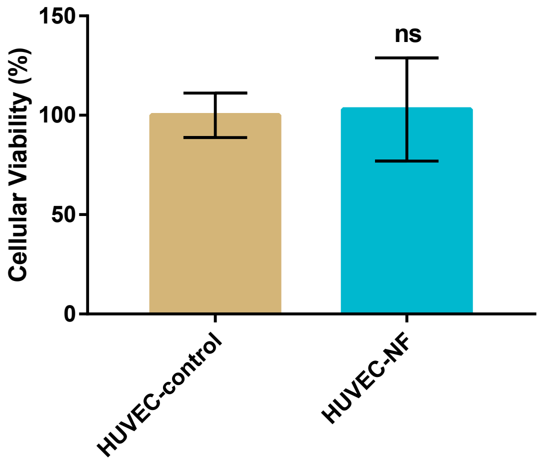

Figure S1. CCK8 assay was used to assess the effect of NF (100 μM, 24 h) on HUVEC cell viability. NF, nuciferin. ns, no significance.

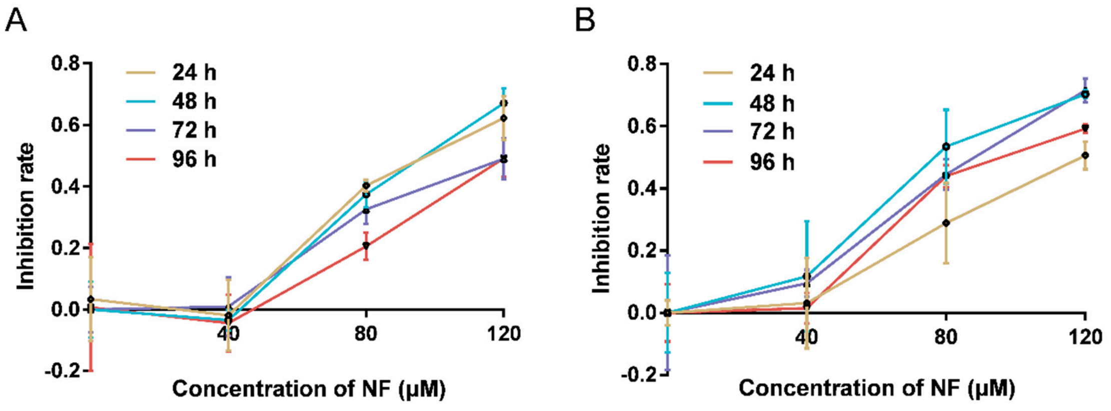

Figure S2. (A, B) CCK8 assay was used to assess inhibition rates in SCC25 and CAL27 cells treated with NF (0~120 μM, 0~96 h). NF, nuciferin.
